# Supplementary material for: GLP-1R Agonists and Their Therapeutic Potential in Inflammatory Bowel Disease and Other Immune-Mediated Inflammatory Diseases, a Systematic Review of the Literature
Source: Biomedicines. 2025 May 6;13(5):1128. doi: 10.3390/biomedicines13051128 (PMC12108654; doi:10.3390/biomedicines13051128)
Supplement: Supplementary file 1 [file biomedicines-13-01128-s001.zip › biomedicines-3586994-supplementary.pdf]

Supplementary Table S1. Animal and in-vitro studies of GLP-RAs on experimental colitis

| Author, Year, Country of publication | Study subjects & models of colitis | Treatment | Results | |
|---|---|---|---|---|
| | | | **What was studied?** | **Effect of liraglutide (DSS + LIG), compared to control (DSS +PBS)** |
| Sun et al, 2024, China (19) | DSS[1]-induced colitis in C57BL/6 mice | Liraglutide 0.6mg/kg/day for 5 days | Body weight (n=8/group), colon length (n=10/group), disease activity index (DAI) | Slower body weight loss, extended colon length, lower disease activity index |
| | | | Histological score, goblet cell loss and crypt distortion (n=4/group) | Attenuated histological score & less goblet cell loss |
| | | | Neutrophil, eosinophil, T-regulatory cells count by flow cytometry (DSS+PBS: n=6, DSS+LIG: n=9) | Dramatic decrease in percentage of eosinophils, but not neutrophils (This effect is not seen in *Rorc* $^{gfp/gfp}$ mice, which lack ILC3s[2].)<br>No significant change in T Reg count. |
| | | | Expression of IL-22, IL-17A, GM-CSF[3], RORγt[4] (DSS+PBS: n=7, DSS+LIG: n=8), NKp46[5] and CCR6 (n=8/group) from large intestinal lamina propria lymphocytes analysed by flow cytometry | Proportion of IL-22 producing ILC3s significantly greater<br>No difference in expression of GM-CSF and IL-17 in ILC3s<br>Marked increase in the % of NKp46 ILC3s and a decrease in %of CCR6 ILC3s. |
| | | | Serum IL-22 and IL-1 β by ELISA[6] (n=3/group) | Increased serum IL-22 levels, no difference in IL-1 β |
| | | | mRNA expression of Ltbr[7], Il18, RegIIIbeta[8], Glp1r, Tjp1[9] and Il23 in large intestinal IEC[10]s of *Rag1* $^{-/-}$ mice was analysed by real-time RT-PCR[11] (n=5/group) | Significant increase in expression of Ltbr[7], Il18 and RegIIIbeta<br>No significant increase in expression of GLP1r, Tjp1 and Il23 |
| | | | GLP-1 receptor protein and tight junction markers (claudin-1 and ZO-1) expression | Upregulated expression of claudin-1 and ZO-1 in intestinal epithelial layer |

[1] Dextran sodium sulfate (DSS), [2]Innate lymphoid cells (ILCs), [3] Granulocyte-macrophage colony-stimulating factor (GM-CSF), [4] Retinoic acid-related orphan receptor (ROR) γt, [5]NKp46 – A natural cytotoxicity receptor, [6]enzyme-linked immunosorbent assay (ELISA), [7]Lymphotoxin beta receptor (Ltbr), [8] regenerating islet-derived protein 3 (RegIII), [9]Tjp1- gene encoding the tight junction protein ZO-1, [10] Intestinal epithelial cells, [11] Real time (reverse transcription) polymerase chain reaction.

| | | | | |
|---|---|---|---|---|
| | | | in colon tissue examined by immunohistochemistry (IHC). | |
| | | | Gut microbiota, using 16S rRNA gene sequencing on faecal samples (n=5/group) | Phylum level: the proportions of *Firmicutes* and *Proteobacteria* were increased.<br>Genus level: increased abundance of Lactobacillus, *Helicobacter*, *Turicibacter* and *Alistipes* but a decreased abundance of *Staphylococcus* and *Faecalibaculum*<br>Species level: elevated abundance of specific microbes such as *Lactobacillus reuteri, Lactobacillus johnsonii* and *Helicobacter typhi* |
| | | | Functional metabolites (n=5/group) | Comparative metabolomics analysis indicated that GLP-1RAs altered faecal & colon metabolic profiles.<br>Pathway enrichment analysis showed that sphingolipid signalling pathway & sphingolipid metabolism were implicated in both faecal & colon metabolic pathways.<br>GLP-1RA improved relative abundance of DMS[12] |
| Yusta et al, 2015, Canada (20) | DSS-induced colitis in C57BL/6 mice | Exendin-4 (Ex-4) | **What was studied?** | **Results** |
| | | | Expression of Glp1r in murine intestinal IELs[13] with RT-PCR (n=6 and 3 independent preparations for small intestine and colon IELs, respectively) | Glp1r transcript is expressed in murine intestinal IELs, and is expressed in RNA from both T αβ and T γδ IELs |
| | | | Cytokine expression in intestinal IELs | Ex-4 significantly attenuated the mRNA and protein expression of proinflammatory cytokines (IL-2, IL-17a, IFNγ & TNFα) in IELs activated by immobilised anti-CD3 and soluble anti-CD28 antibodies. |
| | | | Comparing severity of colitis in Glp1r -/- versus Glp1r +/+ mice (n=9-12 mice/group) | Glp1r -/- mice lost significantly more weight and increased disease activity scores & greater epithelial damage. |
| | | | Expression of genes important for epithelial repair, barrier function, and immune regulation in the colon of Glp1r-/- and Glp1r+/+ mice in the presence or absence of DSS-induced colitis (n=8-12 mice/group) | Even in the absence of DSS, Glp1r-/-mice exhibited significant reductions in expression of genes that contribute to epithelial protection and repair (Tff[14]-1 and -2, Tgf[15]-β1 and -3, Egfr[16], Fgf7[17], Hgf[18]), the innate immune response (Il6, Il1β), inflammation (Il12b) |

[12] N,N-dimethylsphingosine, [13] Intestinal intraepithelial lymphocytes (IELs), [14] Trefoil factor (TFF), [15]Transforming growth factor (TGF), [16]epidermal growth factor receptor (Egfr), [17]keratinocyte growth factor (Fgf7),[18] hepatocyte growth factor (Hgf)

<table>
<tr><td></td><td></td><td></td><td>Expression of immunomodulatory and antimicrobial genes in murine small intestine, by assessing mRNA using RT-qPCR (n=5-6 mice/group)<br><br>Gut microbiota in Glp1r -/- versus Glp1r +/+ mice, by analysing 16S bacterial rRNA genes in the faeces and metagenomic analysis (n=3-5 mice/group)</td><td>Ex-4 markedly upregulated levels of<br><br>1. immunomodulatory genes: Il1b, Il6, Il22, Il12b, Tnfa, Ccl2, Cxcl1, and Cxcl2<br>2. antimicrobial genes: RegIIIγ and RegIIIβ<br>3. genes that play a role in pathogen clearance: IL-5, IL-13.<br><br>Highly significant differences in the relative abundance of Gammaproteobacteria, Bacteroidetes, Firmicutes, and Actinobacteria were detected.</td></tr>
<tr><td>Wong et al, 2022, Canada (25)</td><td>Anti-CD3 antibody induced colitis and LPS[19] induced colitis in C57BL/6J and Lck-Cre mice</td><td>Semaglutide, Exendin-4</td><td colspan="2">Gut IEL GLP-1R is essential for the full effects of GLP-1RAs on gut microbiota, by comparing faecal microflora in $Glp1r^{T\ cell\ +/+}$ and $Glp1r^{T\ cell\ -/-}$ mice (n=8-11).<br><br>In anti-CD3 induced colitis (n=5-10), exendin-4 reduced levels of multiple plasma cytokines (IFNγ, IL-12, IL-1b, IL-2, IL-4, IL-5, IL-6, CXCL1, TNF$\alpha$) by 30-50%<br><br>Anti-inflammatory actions of GLP-1RAs require the gut IEL GLP-1R to selectively restrain local and systemic T cell-induced, but not LPS-induced, inflammation. Such effects are mediated by the suppression of gut IEL effector functions linked to the dampening of proximal T cell receptor signalling in a protein-kinase-A-dependent manner.</td></tr>
<tr><td>Mahdy et al, 2024, Egypt (26)</td><td>Acetic acid (AA) induced colitis in Sprague-Dawley rats (total n=56)</td><td>Dulaglutide (50, 100 and 150 microg/kg)</td><td colspan="2"><table>
<tr><th>What was studied?</th><th>Effect of acetic acid to colons</th><th>Effect on adding Dula (dulaglutide) (50, 100 and 150 µ/kg), compared to acetic acid group</th></tr>
<tr><td>Colon macroscopic scores</td><td>Colons showed erythema, thickness, necrosis and oedema</td><td>Less pronounced macroscopic changes (50 µ/kg slighter effect compared to 100 & 150 µ/kg)</td></tr>
<tr><td>Colon weight & weight/length ratio</td><td>Augmented wet weight & weight/length by 1.40 times (p<0.05)</td><td>Significant reduction in dose dependent manner (5.4%, 16.3%, 21.06% with 50, 100, and 150 µ/kg of dulaglutide respectively)</td></tr>
<tr><td>Colon histopathological changes</td><td>Submucosal massive infiltration of chronic inflammatory cells with development of lymphoid follicles, gland hyperplasia and goblet cell death</td><td>Dula (Dulaglutide) 50 µ/kg: glandular hyperplasia, loss of goblet cells, localised ulceration & significant inflammation.<br>Dula 100 µ/kg: glandular hyperplasia, healed ulcer with minimal inflammation, decrease in goblet cells.<br>Dula 150 µ/kg: glandular hyperplasia, no ulceration, no goblet cell death</td></tr>
</table></td></tr>
</table>

[19] Lipopolysaccharide (LPS)

| | | | | | |
|---|---|---|---|---|---|
| | | | Oxidant/ antioxidant balance: by measuring serum TAC[20] and colonic levels of MDA[21], GSH[22] and SOD[23] | Significantly reduced ($p<0.05$) TAC (51.33%), GSH (77.46%), SOD (49.16%); MDA levels 4.39 times higher ($p<0.05$) | See table below; All $p<0.05$ |
| | | | Colonic NFκB[24] expression using IHC analysis and optical densities determined using Image-Pro Plus program. | Marked increase in positively stained crypts; Substantially higher NFκB scores | Positive brown intensity markedly lowered & was observed in few crypts in Dula 150 µ/kg; Significant reduction in NFκB scores in Dula 100 & 150 µ/kg |
| | | | Colonic IL-6 expression using IHC analysis and optical densities determined using Image-Pro Plus program. | Sizable increased IL-6 positive cells | Reduction in number of IL-6 positive cells |
| | | | Colonic levels of GLP-1, TFF-3 & TGF- β 1 using ELISA<br><br>Colonic levels of PI3K[25], AKT[26] & IFN γ using spectrophotometry<br><br>Serum levels of LDH[27] & CRP[28] | See table below | See table below |

| | TAC | GSH | SOD | MDA |
|---|---|---|---|---|
| 50 µ/kg | +1.46 times | +1.66 times | +1.26 times | -41.69% |
| 100 µ/kg | +1.73 times | +2.22 times | +1.40 times | -51.80% |
| 150 µ/kg | +1.99 times | +2.84 times | +1.66 times | -62.07% |

All $p<0.05$

| | AA compared to control | Treatment group compared to AA | | |
|---|---|---|---|---|
| | | Dula 50 µ/kg | Dula 100 µ/kg | Dula 150 µ/kg |
| GLP-1 | Reduction of 74.91% | Increased by 1.75-fold | Increased by 2.63-fold | Increased by 3.16-fold |
| TFF-3 | Reduction of 65.02% | Increased by 1.07-fold | Increased by 1.68-fold | Increased by 2.07-fold |
| TGF- β 1 | Increased by 7.07-fold | Not significantly reduced | Reduced by 45.64% | Reduced by 58.12% |
| PI3K | Increased by 3.67 times | Reduced by 21.54% | Reduced by 49.16% | Reduced by 59.86% |

[20] Total antioxidant capacity (TAC), [21] malondialdehyde (MDA), [22] reduced glutathione (GSH) [23] superoxide dismutase (SOD) [24] Nuclear factor kappa B (NF-κB) [25] phosphatidylinositol-3-kinase (PI3K) [26] protein kinase B (AKT), [27] lactate dehydrogenase (LDH) , [28]C-reactive protein (CRP)

| | | | | | | | |
|---|---|---|---|---|---|---|---|
| | | | AKT | Increased by 2.77 times | Reduced by 30.17% | Reduced by 43.87% | Reduced by 49.53% |
| | | | IFN γ | Increased by 6.10 folds | Reduced by 28.90% | Reduced by 51.25% | Reduced by 60.45% |
| | | | LDH | Increased by 2.99 times | Reduced by 35.59% | Reduced by 44.44% | Reduced by 50.42% |
| | | | CRP | Increased by 9.88 times | Reduced by 63.39% | Reduced by 75.62% | Reduced by 81.23% |
| | | | All p<0.05 unless stated otherwise. | | | | |

| | | | |
|---|---|---|---|
| Al-Dwairi et al, 2018, Jordan (24) | BALB/c Mouse (total n=20) CSMC[29] stimulated with LPS | Exendin-4 | IHC showed that GLP-1R is expressed in CSMCs[29.]<br>GLP-1RA significantly reduced the expression of TNF-α, IL-1α, TCA-3[30], SDF-1[31], and M-CSF[32] detected by antibody array membrane, ELISA, and real-time PCR.<br>LPS increased NF-κB phosphorylation, while exendin-4 significantly reduced levels of NF-κB phosphorylation.<br>Ex-4 increased CSMC cAMP[33] levels in a dose-dependent fashion |
| Anbazhagan et al, 2016, USA (22) | DSS colitis in C57BL/6J mice (n=10/group) | GLP-1-SSM[34] | GLP-1 SSM partially abrogated DSS induced weight loss and partly restored stool consistency.<br>GLP-1 SSM increasing goblet cells and preserving intestinal epithelial architecture in DSS induced colitis.<br>GLP-1 SSM markedly alleviated colitis by reducing expression of pro-inflammatory IL-1β (mRNA levels measured with real-time PCR).<br>Diarrhoea was alleviated by increasing protein expression of intestinal chloride transporter DRA, detected with immunofluorescence staining of colonic sections. |
| Bang-Berthelsen, 2016, Denmark (23) | AdTR[35] colitis model of Mice (CB-17 SCID and BALB/c) & in vitro (n=75 (27 non-IBD controls, 17 Crohn's, 31 | 0.6m mg/kg Liraglutide | Liraglutide upregulated IL-33, mucin 5b, and CCL20 in murine Brunner's glands, measured by qRT-PCR on complementary DNA.<br>Prophylactic treatment with liraglutide improves disease [36]in AdTr colitis.<br>IL-33, GLP-1R, and CCL20 are deregulated in human IBD, measured by qRT-PCR. |

[29] Colon smooth muscle cells (CSMCs), [30] T cell activation gene-3 (TCA-3), [31]stromal cell-derived factor-1 (SDF-1), [32] macrophage colony stimulating factor (M-CSF, [33] cyclic adenosine monophosphate (cAMP), [34] sterically stabilised phospholipid micelles (SSM), [35]T cell driven adoptive transfer (AdTr), [36] Colon weight: length ratios, histopathological score by evaluating severity of lesion, hyperplasia, crypt damage and percentage area involved

<table>
<tr><td></td><td>ulcerative colitis).</td><td></td><td></td></tr>
<tr><td>Kato, 2021, Japan (27)</td><td>DSS colitis in mice</td><td>Liraglutide</td><td>
<table>
<tr><td>What was studied?</td><td>Results</td></tr>
<tr><td>Relative expression levels of gut bacterium in caecal contents using quantitative PCR, following single liraglutide injection followed by 16h fast. (control n=7, liraglutide n=7)</td><td>Phylum level:<br>- significantly decreased Bacteroidetes and<br>- tended to increase Actinobacteria.<br>- Firmicutes and Proteobacteria were not changed.<br><br>Genus level: significantly reduced Ruminococcus and no significant changes for Akkermansia</td></tr>
<tr><td>Effect of liraglutide on gene expression levels of bacterial proteins that could affect host metabolism</td><td>Expression of FTHFS[37] significantly increased.<br>Expression of BCoAT[38] significantly decreased.<br>Increased expression of ClpB[39] by 400x (control n=7, lira n=7)</td></tr>
<tr><td>Does ClpB expression relate to E. coli number (n=14)</td><td>Significant positive correlation of E.coli 16S rRNA expression with ClpB</td></tr>
<tr><td>Whether sympathetic nervous system activation was involved – by examining levels of intestinal norepinephrine (NE)</td><td>Liraglutide significantly increased NE[41]` in plasma & cecal contents.<br>Medetomidine[40] did not increased ClpB expression in cecal contents (saline control, n=7, saline lira n=6, medetomidine control n=5, medetomidine lira n=5) & NE levels in plasma & cecal contents (saline control, n=4, saline lira n=5, medetomidine control n=5, medetomidine lira n=8).<br>Similar findings on adrenalectomized mice – indicate that NE in cecal contents could be derived from sympathetic nerve terminals.</td></tr>
<tr><td>Effects of liraglutide on tight junction gene expression in caecum</td><td>
<table>
<tr><td>mRNA levels in caecum</td><td>Normal conditions (control n=6-8; liraglutide n=5-8)</td><td>DSS colitis (control n=7, liral n=9)</td></tr>
<tr><td>Occludin</td><td>No alteration</td><td>Significantly lower</td></tr>
<tr><td>TNF-$\alpha$</td><td>No alteration</td><td>Significantly higher</td></tr>
<tr><td>RegIII b</td><td>Significantly increase</td><td>Tended to be higher</td></tr>
<tr><td>IL-33</td><td>Significantly increase</td><td>Tended to be higher</td></tr>
</table>
</td></tr>
<tr><td>Effects of liraglutide on caecal mRNA levels, on TLR4[42] (receptor for LPS)</td><td>Caecal contents of ClpB increased.</td></tr>
</table>
</td></tr>
</table>

[37] formate–tetrahydrofolate ligase (FTHFS), which is related to acetic acid synthesis.
[38] butyryl-CoA: acetate CoA-transferase (BCoAT), which is related to butyrate synthesis.
[39] Caseinolytic protease B (ClpB), a protein component of E. coli
[40] Medetomidine, an *α2 receptor agonist which suppresses SNS.*
[41] NE: Norepinephrine

| | | | | |
|---|---|---|---|---|
| | | | knockout mice in DSS colitis (control n=5, liraglutide n=4) | Caecal occludin & TNF α not different between liraglutide group and control group – suggesting liraglutide induced reduction in occludin gene expression levels was LPS- and TLR4- dependent.<br>Caecal RegIII b significantly higher & IL-33 levels higher but p=0.091 – suggesting liraglutide induced increase in these genes was LPS & TLR4 independent. |
| | | | Effects of liraglutide on bacterial translocation (BT) in DSS colitis | Cecal occludin mRNA levels negatively correlated with ClpB in cecal contents (total n=18, liraglutide n=11).<br>In wildtype mice, Rate of BT 100% (9/9) in liraglutide group (n=9); 14.3% (1/7) in control group (n=7).<br>In TLR4KO mice, rate of BT 100% in lira group (n=-4); 80% in control group (n=5).<br>Cecal occludin mRNA sig lower in BT-positive group than BT-negative group (wildtype BT-positive n=6, wildtype BT-negative n=10, TLR4KO BT positive n=8). |
| Biagioli, 2022, Italy (28) | In vitro (n=7 Crohn's disease) and three mouse (BALB/c and C57BL/6NCrl) models of colitis (TNBS[41], oxazolone and DSS) | Liraglutide | Liraglutide did not interfere with anti-inflammatory effects exerted by BAR501 (GPBAR1 agonist), as measured by assessing expression levels of IL-8, IL-1b and CCL2 mRNA by real-time PCR in HT29 cells (human colorectal adenocarcinoma).<br><br>Liraglutide increased ACE2 mRNA expression in HT29 cells, in HT29 and U937 (human monocyte cell line) cells co-cultures stimulated with LPS. | |

[42] TLR4= Toll like receptor4
[43] Trinitrobenzenesulfonic acid (TNBS),
